# Supplementary material for: Complete chloroplast genomes of Zingiber montanum and Zingiber zerumbet: Genome structure, comparative and phylogenetic analyses
Source: PLoS One. 2020 Jul 31;15(7):e0236590. doi: 10.1371/journal.pone.0236590 (PMC7394419; doi:10.1371/journal.pone.0236590)
Supplement: S1 Table — (DOCX) [file pone.0236590.s001.docx]

**S1Table. Features of the chloroplast genomes of *Z. montamum* and *Z. zerumbet*.**

| **Species** | **Regions** | **Positions** | **Length (bp)** | **T/U (%)** | **C (%)** | **A (%)** | **G (%)** | **AT/U (%)** |
| --- | --- | --- | --- | --- | --- | --- | --- | --- |
| ***Z. montanum*** | Genome |  | 164,464 | 32.37 | 18.17 | 31.88 | 17.57 | 64.25 |
|  | LSC |  | 87,856 | 33.78 | 17.21 | 32.58 | 16.42 | 66.37 |
|  | IRa |  | 30,356 | 28.91 | 19.52 | 30.57 | 20.99 | 59.48 |
|  | SSC |  | 15,803 | 34.55 | 15.57 | 36.21 | 13.67 | 70.77 |
|  | IRb |  | 30,449 | 28.92 | 19.51 | 30.62 | 20.95 | 59.54 |
|  | Protein coding genes |  | 83,496 | 31.66 | 17.01 | 31.61 | 19.71 | 63.27 |
|  |  | 1st position | 27,832 | 24.01 | 18.14 | 31.56 | 26.29 | 55.57 |
|  |  | 2nd position | 27,832 | 32.55 | 19.78 | 30.45 | 17.23 | 62.99 |
|  |  | 3rd position | 27,832 | 38.43 | 13.12 | 32.83 | 15.62 | 71.26 |
|  | tRNA |  | 2,876 | 25.00 | 23.68 | 22.01 | 29.31 | 47.01 |
|  | rRNA |  | 9,046 | 18.75 | 23.58 | 26.18 | 31.49 | 44.93 |
| ***Z. zerumbet*** | Genome |  | 163,589 | 32.14 | 18.47 | 31.59 | 17.80 | 63.73 |
|  | LSC |  | 89,161 | 33.51 | 17.6 | 32.19 | 16.71 | 65.70 |
|  | IRa |  | 29,393 | 28.92 | 19.75 | 30.06 | 21.27 | 58.98 |
|  | SSC |  | 15,642 | 34.31 | 15.78 | 36.05 | 13.86 | 70.36 |
|  | IRb |  | 29,393 | 28.91 | 19.75 | 30.06 | 21.27 | 58.98 |
|  | Protein coding genes |  | 84,042 | 31.56 | 17.18 | 31.49 | 19.77 | 63.05 |
|  |  | 1st position | 28,014 | 23.85 | 18.14 | 31.49 | 26.52 | 55.35 |
|  |  | 2nd position | 28,014 | 32.51 | 20.1 | 30.11 | 17.29 | 62.61 |
|  |  | 3rd position | 28,014 | 38.32 | 13.3 | 32.88 | 15.50 | 71.20 |
|  | tRNA |  | 2,877 | 24.92 | 23.77 | 22.00 | 29.30 | 46.92 |
|  | rRNA |  | 9,046 | 18.70 | 23.59 | 26.09 | 31.62 | 44.79 |
