# Supplementary material for: Complete chloroplast genomes of Zingiber montanum and Zingiber zerumbet: Genome structure, comparative and phylogenetic analyses
Source: PLoS One. 2020 Jul 31;15(7):e0236590. doi: 10.1371/journal.pone.0236590 (PMC7394419; doi:10.1371/journal.pone.0236590)
Supplement: S3 Table — (DOCX) [file pone.0236590.s003.docx]

**S3 Table. Genes with introns in the chloroplast genomes of *Z. montanum* and *Z. zerumbet*.**

| **Species** | **Gene** | **Location** | **Exon І (bp)** | **Intron І (bp)** | **Exon ІІ (bp)** | **Intron ІІ (bp)** | **Exon ІІІ (bp)** |
| --- | --- | --- | --- | --- | --- | --- | --- |
| ***Z. montanum*** | *trnA-UGC* | IR | 35 | 800 | 38 |  |  |
|  | *trnI-GAU* | IR | 35 | 935 | 42 |  |  |
|  | *trnG-GCC* | LSC | 14 | 716 | 48 |  |  |
|  | *trnK-UUU* | LSC | 35 | 2683 | 37 |  |  |
|  | *trnL-UAA* | LSC | 35 | 533 | 50 |  |  |
|  | *trnV-UAC* | LSC | 37 | 620 | 38 |  |  |
|  | *accD* | LSC | 934 | 288 | 620 |  |  |
|  | *rps12 ** | LSC/IR | 114 | - | 27 | 540 | 231 |
|  | *rps16* | LSC | 212 | 735 | 40 |  |  |
|  | *rpl2* | IR | 385 | 650 | 443 |  |  |
|  | *rpl16* | LSC | 402 | 1067 | 9 |  |  |
|  | *petB* | LSC | 6 | 820 | 642 |  |  |
|  | *petD* | LSC | 8 | 741 | 481 |  |  |
|  | *atpF* | LSC | 425 | 796 | 145 |  |  |
|  | *ndhA* | SSC | 518 | 1093 | 562 |  |  |
|  | *ndhB* | IR | 789 | 667 | 777 |  |  |
|  | *rpoC1* | LSC | 1632 | 724 | 432 |  |  |
|  | *clpP* | LSC | 255 | 633 | 291 | 834 | 69 |
|  | *ycf3* | LSC | 153 | 808 | 228 | 710 | 132 |
| ***Z. zerumbet*** | *trnA-UGC* | IR | 35 | 801 | 38 |  |  |
|  | *trnI-GAU* | IR | 35 | 934 | 42 |  |  |
|  | *trnG-GCC* | LSC | 14 | 712 | 48 |  |  |
|  | *trnK-UUU* | LSC | 35 | 2606 | 37 |  |  |
|  | *trnL-UAA* | LSC | 35 | 519 | 50 |  |  |
|  | *trnV-UAC* | LSC | 37 | 616 | 38 |  |  |
|  | *accD* | LSC | 851 | 177 | 643 |  |  |
|  | *rps12** | LSC/IR | 114 | - | 27 | 540 | 231 |
|  | *rps16* | LSC | 212 | 745 | 40 |  |  |
|  | *rpl2* | IR | 443 | 650 | 385 |  |  |
|  | *rpl16* | LSC | 402 | 1043 | 9 |  |  |
|  | *petB* | LSC | 6 | 784 | 642 |  |  |
|  | *petD* | LSC | 8 | 740 | 481 |  |  |
|  | *atpF* | LSC | 425 | 807 | 145 |  |  |
|  | *ndhA* | SSC | 518 | 1073 | 562 |  |  |
|  | *ndhB* | IR | 789 | 667 | 777 |  |  |
|  | *rpoC1* | LSC | 1632 | 723 | 432 |  |  |
|  | *clpP* | LSC | 255 | 613 | 291 | 837 | 69 |
|  | *ycf3* | LSC | 153 | 808 | 228 | 704 | 132 |

* The *rps12* gene is divided into 5′-*rps12* in the LSC region and 3′-*rps12* in the IR region.
