# Supplementary material for: Complete chloroplast genomes of Zingiber montanum and Zingiber zerumbet: Genome structure, comparative and phylogenetic analyses
Source: PLoS One. 2020 Jul 31;15(7):e0236590. doi: 10.1371/journal.pone.0236590 (PMC7394419; doi:10.1371/journal.pone.0236590)
Supplement: S1 Fig — Ψ, pseudogenes. Boxes above the main line indicate the adjacent border genes. The figure is not to scale with respect to sequence length and shows relative changes only at or near the IR/SC borders. (DOCX) [file pone.0236590.s011.docx]

**LSC**

**IRa**

**IRb**

**SSC**

**LSC**

30,356 bp

15,803 bp

30,449 bp

89,161 bp

29,393 bp

15,642 bp

29,393 bp

88,460 bp

25,451 bp

16,528 bp

25,451 bp

*Ψycf1*

*ndhF*

*ycf1*

*trnH*

*rpl22*

*rps19*

*Ψycf1*

*ndhF*

*ycf1*

*rps19*

*psbA*

85 bp

157 bp

8 bp

31 bp

1525 bp

3921 bp

157 bp

114 bp

125 bp

924 bp

33 bp

4789 bp

665 bp

*ycf2*

*trnM*

256 bp

*psbA*

205 bp

87,856 bp

***Z. spectabile***

**JX088661**

***Z. montanum***

**MK262727**

***Z. zerumbet***

**MK262726**

*rpl22*

*rps19*

59 bp

140 bp

*Ψycf1*

*ndhF*

*ycf1*

*rps19*

*psbA*

8 bp

31 bp

3987 bp

1519 bp

3987 bp

124 bp

138 bp

3921 bp

***Z. officinale***

**NC_044775**

*psbA*

87,486 bp

29,779 bp

15,577 bp

29,779 bp

*rpl22*

20 bp

*rps19*

*Ψycf1*

*ndhF*

*ycf1*

*rps19*

149 bp

3336 bp

9 bp

1554 bp

3900 bp

148 bp

112 bp

563 bp

**S1 Fig. Comparison of the borders of the LSC, SSC, and IR regions among four *Zingiber* species chloroplast genomes.** Ψ, pseudogenes. Boxes above the main line indicate the adjacent border genes. The figure is not to scale with respect to sequence length, and only shows relative changes at or near th IR/SC borders.
