# Supplementary material for: Complete chloroplast genomes of Zingiber montanum and Zingiber zerumbet: Genome structure, comparative and phylogenetic analyses
Source: PLoS One. 2020 Jul 31;15(7):e0236590. doi: 10.1371/journal.pone.0236590 (PMC7394419; doi:10.1371/journal.pone.0236590)
Supplement: S2 Fig — Ka, nonsynonymous; Ks, synonymous; Zm, Z. montanum; Zo, Z. officinale; Zs, Z. spectabile; Zz, Z. zerumbet. (DOCX) [file pone.0236590.s012.docx]

**
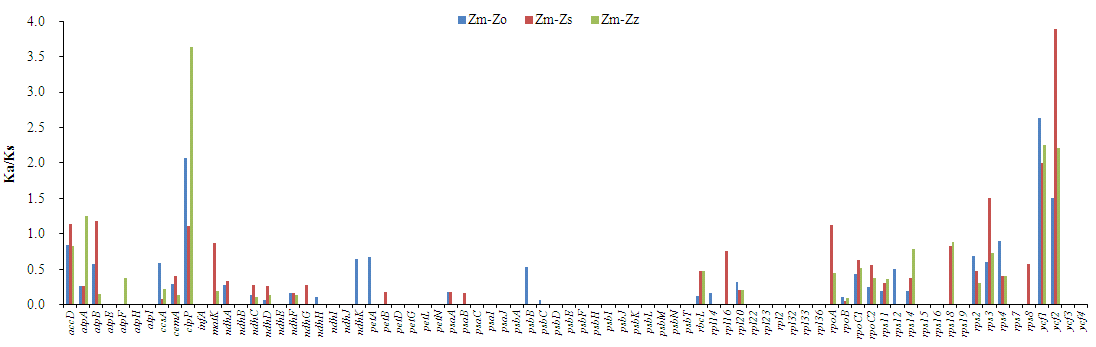
**

**S2 Fig. Ka/Ks ratios of 78 protein-coding genes of the *Z. montanum* chloroplast genome vs. three *Zingiber* species.** Ka, nonsynonymous; Ks, synonymous; *Zm*, *Z. montanum*; *Zo*, *Z. officinale*; *Zs*, *Z. spectabile*; *Zz*, *Z. zerumbet*.
