# Supplementary material for: Complete chloroplast genomes of Zingiber montanum and Zingiber zerumbet: Genome structure, comparative and phylogenetic analyses
Source: PLoS One. 2020 Jul 31;15(7):e0236590. doi: 10.1371/journal.pone.0236590 (PMC7394419; doi:10.1371/journal.pone.0236590)
Supplement: Attachment — Submitted filename: response to reviewer2.doc [file pone.0236590.s014.doc]

Reviewers' comments:

Reviewer's Responses to Questions

**Comments to the Author**

1. If the authors have adequately addressed your comments raised in a previous round of review and you feel that this manuscript is now acceptable for publication, you may indicate that here to bypass the “Comments to the Author” section, enter your conflict of interest statement in the “Confidential to Editor” section, and submit your "Accept" recommendation.

Reviewer #2: All comments have been addressed

2. Is the manuscript technically sound, and do the data support the conclusions?

The manuscript must describe a technically sound piece of scientific research with data that supports the conclusions. Experiments must have been conducted rigorously, with appropriate controls, replication, and sample sizes. The conclusions must be drawn appropriately based on the data presented.

Reviewer #2: Yes

3. Has the statistical analysis been performed appropriately and rigorously?

Reviewer #2: Yes

4. Have the authors made all data underlying the findings in their manuscript fully available?

The [PLOS Data policy](http://www.plosone.org/static/policies.action#sharing) requires authors to make all data underlying the findings described in their manuscript fully available without restriction, with rare exception (please refer to the Data Availability Statement in the manuscript PDF file). The data should be provided as part of the manuscript or its supporting information, or deposited to a public repository. For example, in addition to summary statistics, the data points behind means, medians and variance measures should be available. If there are restrictions on publicly sharing data—e.g. participant privacy or use of data from a third party—those must be specified.

Reviewer #2: Yes

5. Is the manuscript presented in an intelligible fashion and written in standard English?

PLOS ONE does not copy edit accepted manuscripts, so the language in submitted articles must be clear, correct, and unambiguous. Any typographical or grammatical errors should be corrected at revision, so please note any specific errors here.

Reviewer #2: Yes

6. Review Comments to the Author

Please use the space provided to explain your answers to the questions above. You may also include additional comments for the author, including concerns about dual publication, research ethics, or publication ethics. (Please upload your review as an attachment if it exceeds 20,000 characters)

Reviewer #2: I only have a few questions about this revision:

I am not sure if I am allowed to comment on the authors' response to the other reviewer's question, but I saw such statement: "... identifies some interspecific species at genus-level ..." I simply never heard of "interspecific species".
Response: Sorry, we used the wrong words “"interspecific species” in the statement. The correct words should be “identifies some interspecific relationships at genus-level”.

Figure 4A legend: Please add a sentence explaining that genes with zero SNP were not shown.

Response: Yes, we agree. We add the sentence that the genes with zero SNP were not shown in Figure 4A.

Line 62-64: The sentence is confusing. I think "X is limited in high-resolution identification" means X markers can only be used to resolve the relationship among species within the same genus, but not the relationship among genus or families? Is this what the authors want to say? If true, is the main purpose of this study to resolve higher level taxonomy? Please clarify this. The fact that I, the other reviewer, and your English editor all are confused by this sentence suggests this needs to be rephrased.

Response: We revised the lines 62-64 as follows:

These analyses have succeeded in clarifying the phylogenetic relationships and degrees of variation among *Zingiber* species, but in general have been limited in breadth of resolution.

7. PLOS authors have the option to publish the peer review history of their article ([what does this mean?](https://journals.plos.org/plosone/s/editorial-and-peer-review-process#loc-peer-review-history)). If published, this will include your full peer review and any attached files.


If you choose “no”, your identity will remain anonymous but your review may still be made public.


**Do you want your identity to be public for this peer review?** For information about this choice, including consent withdrawal, please see our [Privacy Policy](https://www.plos.org/privacy-policy).

Reviewer #2: No

[NOTE: If reviewer comments were submitted as an attachment file, they will be attached to this email and accessible via the submission site. Please log into your account, locate the manuscript record, and check for the action link "View Attachments". If this link does not appear, there are no attachment files.]

While revising your submission, please upload your figure files to the Preflight Analysis and Conversion Engine (PACE) digital diagnostic tool, <https://pacev2.apexcovantage.com/>. PACE helps ensure that figures meet PLOS requirements. To use PACE, you must first register as a user. Registration is free. Then, login and navigate to the UPLOAD tab, where you will find detailed instructions on how to use the tool. If you encounter any issues or have any questions when using PACE, please email PLOS at [figures@plos.org](mailto:figures@plos.org). Please note that Supporting Information files do not need this step.

Response: We used PACE to change the figure files to meet PLOS requirements one by one.
